# Biodegradation of total petroleum hydrocarbons from acidic sludge produced by re-refinery industries of waste oil using in-vessel composting

**DOI:** 10.1186/s40201-017-0267-1

**Published:** 2017-02-27

**Authors:** Alireza Asgari, Ramin Nabizadeh, Amir Hossein Mahvi, Simin Nasseri, Mohammad Hadi Dehghani, Shahrokh Nazmara, Kamyar Yaghmaeian

**Affiliations:** 1 Center for Solid Waste Research (CSWR), Institute for Environmental Research (IER), Tehran University of Medical Sciences (TUMS), Tehran, Iran; 2 Department of Environmental Health Engineering, School of Public Health, Tehran University of Medical Sciences, Tehran, Iran; 3 Center for Water Quality Research (CWQR), Institute for Environmental Research (IER), Tehran University of Medical Sciences (TUMS), Tehran, Iran

**Keywords:** Acidic sludge, Re-refinery industry, Aerated in-vessel composting, Total petroleum hydrocarbons, Biodegradation, Kinetic model

## Abstract

**Background:**

In Iran, re-refinery industry has been developed many years ago based on the acid-clay treatment. Acidic sludge with high concentration of total petroleum hydrocarbon (TPH) is the final products of some facilities. In this study removal of TPH by aerated in-vessel composting was investigated.

**Methods:**

In order to microorganisms seeding and nutrient providing, urban immature compost was added as an amendment to acidic sludge. The ratios of acidic sludge (AS) to compost were, 1:0 (as control), 1:5, 1:8, 1:10, 1:15, 1:20, 1:30, 1:40, 1:50, 1:75 and 1:100 (as dry basis) at a C: N: P ratio of 100:5:1 and 45–65% moisture content for 70 days.

**Results:**

The removal efficiency in all reactors was more than 48%. The highest and lowest TPH removal was observed in 1:5 (71.56%) and 1:100 (48.53%) mixing ratios, respectively. The results of the control reactors showed that biological treatment was the main mechanism for TPH removal. Experimental data was fitted second order kinetic model (*R* ^2^ > 0.8006). Degradation of TPH in 1:5 mixing ratio (k_2_ = 0.0038 gmg ^−1^d^−1^; half-life = 3.08d) was nearly three times faster than 1:100 mixing ratio (k_2_ = 0.0238; half-life = 8.96d). The results of the control reactors showed that biological treatment was the main mechanism for TPH removal.

**Conclusion:**

The results of this study revealed in-vessel composting with immature urban compost as the amendment maybe recommended as an effective method for TPH remediation.

## Background

Every day, million tons of used oil are collected from repair shops and are taken to larger facilities which are called re-refineries and their contaminants are separated [1]. Engine oils contain 90% oil and less than 10% additive materials [2]. Usually, after a vehicle runs for a long period of time, oil becomes degraded and loses its original quality and therefore needs to be replaced [3]. Engine oil, hydraulic oil, lubricants oil, flux oil and wastes such as filter cake and acidic sludge are the final products of some facilities. There are some specific technologies for recycling materials of used oil which contain high concentration of contaminants [4–7]. In Iran, re-refinery industry has been developed since 40 years ago based on the acid-clay treatment. In average from every 100 barrels of refined oil about 15 barrels of acidic sludge are produced. In Iran, nearly 950000 tons of used oil is collected and recycled annually and at the end of this procedure approximately 80000 to 150000 tons of acidic sludge are produced [8, 9]. This waste contains a high concentration of total petroleum hydrocarbon (TPH), volatile organic carbon (VOC), metals and other materials which are added to the oil during the manufacturing process [10, 11]. Oily sludge Produced by Re-refinery Industries contain a material that are considered to be potent carcinogen and immunotoxicant. The acidity of this oily sludge (pH 1.5–3) of is owing to sulphuric acid that was consumed in old wax refining methods in the refinery [12].

TPH is a complex of alkane, aromatics, nitrogen, sulfur and asphaltenes fractions [10, 13]. Intentional leakage or accidental discharge and direct disposal of this type of hazardous waste into the environment, especially soil and water resources have been created great adverse effects on the environment and human health due to various pollutants including volatile hydrocarbons emission into the air and permeation of the contaminants in the groundwater and soil [14]. Composting as an economically and environmentally method of bioremediation has been considered for treating some wastes recently [15–18]. Composting process has been used for biodegradation and removal of PAHs, explosives, chlorophenols compounds and PCBs [19, 20]. Easy to provide the desired composting conditions (temperature, required oxygen, humidity content, mixing ration and pH), low time reaction, low cost, odor control are the advantage of In-vessel composting. Previous studies apparently showed that control of the operational composting parameters is complex and this is a crucial importance during the composting treatment of contaminated wastes [19].

Aerated in-vessel composting is an approach, in which various environmental factors such as moisture content, temperature, oxygen, nutrients, pH and mixing ratio can be easily controlled. In-vessel composting has been used for co-composting of sludge oil with other materials [16, 21–24]. Thus the aim of this study was to use the in-vessel composting process for reducing TPH from the acidic sludge of the re-refineries industries.

## Methods

### Feed preparation

Acidic sludge samples were obtained from re-refinery units of the Eshtehard (south west of Alborz) industrial town in compliance with sampling protocol. Required immature compost was collected from the aeration hall of Tehran composting plant at first week of composting term and in different depths and 3 points. The samples were mixed together and then impure materials such as glass and plastic were separated from mixed samples. Collected samples were crushed to reach a size of 2 cm and were finally mixed with dry manure (5% w/w) as a source of microorganisms.

### Pilot features

Plastic containers (500 ml) with waterproof screw cap were used for in-vessel composting reactor. Each container was joined to an oxygen blower and water supply and also equipped by thermometer. Diaphragm pumps (Model ACO 5505, Hailea, Guangdong, China) and distribution system for adjustable air (the same amount of each container) were used for oxygen supply and effluent air to each container was 20 ml per minute.

### Design of experiments

#### Acidic sludge ratio into compost

Firstly, various ratios of the acidic sludge to compost were provided (1:5, 1:8, 1:10, 1:15, 1:20, 1:30, 1:40, 1:50, 1:75 and 1:100). Secondly, for controlling the process, two reactors, which only contain acidic sludge (acidic sludge to compost ratio was 1:0) were used. Also, HgCl_2_ (2%) was added to one reactor as biocide [21] and another reactor had no additive. These reactors were aerated to determine the reduction of TPH through aeration and volatilization. The total adaptation time for preventing toxic shock was 20 days. Then, the acidic sludge was gradually added to the reactors in four phases, with an interval of 5 days.

#### Nutrients and moisture adjustment

The amount of nitrogen (N) and phosphorus (P) in each reactor were measured after a period of adjustment. Phosphorus, nitrogen and carbon (C) were adjusted about 1:5:100 using NH_4_Cl (99.99%, Sigma-Aldrich, St. Louis, MO) as a source of N and KH_2_PO_4_ (99.55%, Sigma-Aldrich) as a source of P. The moisture content in each reactor was maintained between 45 and 65% in all durations of the composting process.

### Analytical methods

The reactor contents were thoroughly mixed before sampling and samples were obtained from various depths. For preparation of each sample, 4 ml of n- hexane (99.95%, Merck, Darmstadt, Germany) was added to 1 g of dry sample and then was stirred 1 minute at 300 rpm and then settled particles were kept in refrigerator at 4 °C until analyzed. The amount of TPH was measured using GC-FID (Model CP-3800, Varian, Belrose, Australia) according to TNRCC method [21, 25]. For determination of TPH, 0.6 mL of extracted sample was injected into GC-FID and a CP-Sil8CB capillary column (30-m length, 0.32-mm internal diameter and 0.25 μm film thickens). Helium was used as a carrier gas with 11 Psi inlet pressure at a spilt ratio 25% and through-column rate 2.9 mLmin^−1^. The oven temperature program was as follow: 35 °C isothermal for 3 min, 15 °C min^−1^ rate to 300 °C, isothermal for 5 min, and final temperature at 310 °C for 3 min with rate of 200 °C.min^−1^. The injection port and detector temperatures were 280 and 325 °C, respectively. Hydrogen, air, and makeup (nitrogen) flow rates for the FID were 40, 450, and 30mLmin^−1^, respectively. Total run time was 29 min. The calibration curve for the external standard technique was verified each working day. Hydrocarbons measured in this study were C6–C35. Humidity content were determined by digital hygrometer (Model pH-Moisture Meter, China) and the method of drying at 105 °C for 24 h by oven (Model tactical 308, Gallenkamp, Loughborough, UK). The temperature and pH were measured with mercury thermometers and direct pH meter (Model pH Meter, Combi-Tester, TFA, Germany) and laboratory pH meter (Model, Portable Meter, Germany), respectively. For measuring pH at the laboratory, 1 g of dry sample was dissolved at 5 ml distilled water [26] and was agitated for 1 min (200 rpm), then supernatant was taken for pH detection. Organic carbon was determined using a loss-on-ignition method in furnace (Model Muffle Furance, England). To achieve this purpose, 1 g of dried sample was put at 600 °C for 2 h [27]. Total Kjeldahl nitrogen (TKN) was analyzed using Method 4500-N_org_ of standard method. First 1 g of dry sample, 3 mL of 0.1 M sulfuric acid (in order to prevent the escape Ammonia) [28] and 250 to 300 ml of distilled water was completely mixed. The amount of total phosphorus was measured by dissolving 1 g of dried sample in 50 ml of distilled water and then total phosphorous was analyzed according to 4500-C method of standard methods [29]. Metals were determined using the ICP device (Model ARCOS FHE12, Spectro, Kleve, Germany) and digestion process carried out by nitric acid digestion [30]. Phosphorus, nitrogen, organic carbon and TPH were weekly measured and ambient and reactor temperature were monitored daily. pH was determined during reaction time and then 1 time per day.

### Kinetic study

In this study, kinetic analysis (first and second orders) as an important factor for degradation of TPH removal were studied. The mathematical model used to determine the first and second order reactions were LnC_t_ = LnC_0_-k_1_t and 1/C_t_ = k_2_t + 1/C_0_, respectively. Where C_0_ and C_t_ are the concentration of TPH at the initial time and certain time (t), respectively. The first and second order constant are k_1_ and k_2_, respectively. Half-lives (t_1/2_) as the time required consuming half of TPH concentration in time t of in-vessel composting was calculated according to following equations:

$$ \begin{array}{l}{\mathrm{t}}_{1/2}=\mathrm{Ln}2/{\mathrm{k}}_1;\ \mathrm{for}\ \mathrm{first}\ \mathrm{order}\ \mathrm{reactions}\ \mathrm{and}\\ {}{\mathrm{t}}_{1/2}=1/{\mathrm{k}}_2{\mathrm{C}}_0\mathrm{for}\ \mathrm{s}\mathrm{econd}\ \mathrm{order}\mathrm{s}\ \mathrm{reactions}\end{array} $$

## Results

### Characteristics of the acidic sludge and immature compost

The characteristics of the acidic sludge and immature compost used in this research are shown in Table 1. As it clears more than 50% of acidic sludge mass containing by TPH, and pH in this type of sludge is extremely acidic (pH = 1.35).

**Table 1:** Physicochemical properties of the acidic sludge and immature compost

Parameter	Unit	Acidic Sludge	immature Compost
Organic Carbon (OC)	g kg^−1^	542.44	240.55
TKN	g kg^−1^	9.96	4.57
TP	g kg^−1^	2.22	2.10
pH	-	1.35	7.28
Moisture content	%	10.60	45.27
TPH	g kg^−1^	521.12	N.D

The C: N ratio was 54.46:1 for the acidic sludge and 52.63:1 for the immature compost and C: P for the acidic sludge and immature compost were approximately 244.34:1 and 114.55:1 respectively.

Difference between concentrations of the elements in the acidic sludge is shown in Table 2. It is obvious that the acidic sludge contains high concentration of Zn, Cu, Fe, Mo, B with exceeding 1000 mg/kg. Mn, Al, Pb, Cr and Ba were also relatively high (more than 100 mg/kg). The elements, Sr, Li, Ni and Sn were in the range of 40 to 60 mg/kg, but the other elements have a lower concentration “less than 10 mg/kg”.

**Table 2:** Elemental analysis of the acidic sludge used in this study

Element	Unit	Concentration	Element	Unit	Concentration
Zn	mg kg^−1^	12248.7	V	mg kg^−1^	8.28
Cu	mg kg^−1^	4420.46	Ag	mg kg^−1^	6.18
Fe	mg kg^−1^	4033.58	As	mg kg^−1^	4.96
Mo	mg kg^−1^	2269.54	Cd	mg kg^−1^	4.44
B	mg kg^−1^	1369.36	Co	mg kg^−1^	3.78
Mn	mg kg^−1^	398.06	Hg	mg kg^−1^	3.49
Al	mg kg^−1^	273.02	Sb	mg kg^−1^	2.88
Pb	mg kg^−1^	132.7	Se	mg kg^−1^	0.14
Cr	mg kg^−1^	119.09	Tl	mg kg^−1^	0.13
Ba	mg kg^−1^	110.33	Be	mg kg^−1^	0.01
Sn	mg kg^−1^	58.65	Li	mg kg^−1^	47.92
Ni	mg kg^−1^	56.01	Sr	mg kg^−1^	43.33

### TPH removal in composting reactors

The residual of TPH during adjustment period and the period of composting in 10 weeks in each of the reactors (with various mixing ratios) are shown in Table 3. The maximum and minimum concentration of TPH in a period of adaptation with the sludge to compost ratio of 1:5 and 1:100 were 104.22 and 5.21 g.kg^−1^ and after 10 weeks, nearly 83.93 and 2.80 g of TPH were removed. The minimum removal efficiency of the biodegradation process in 10 weeks were 48% in all reactors. On the other hand, the maximum removal percentage have occurred in the ratios of 1:5 (71.56%) and 1:8 (70.58%).

**Table 3:** TPH removal efficiency during composting period

Time (week)	The residual TPH (mg.kg^−1^)									
	1:5 ratio	1:8 ratio	1:10 ratio	1:15 ratio	1:20 ratio	1:30 ratio	1:40 ratio	1:50 ratio	1:75 ratio	1:100 ratio
Adjustment period	104.22	65.14	52.11	34.74	26.06	17.37	13.03	10.42	6.95	5.21
0	85.41	54.64	44.97	30.72	22.94	15.44	11.86	9.24	6.42	4.69
2	62.37	39.87	40.87	23.16	16.82	11.83	9.90	8.60	5.98	3.90
3	31.38	21.25	20.17	25.56	12.97	9.12	7.32	5.93	4.30	2.98
4	29.01	21.16	21.29	15.81	12.49	9.19	7.10	5.89	4.06	3.01
5	26.65	17.91	17.34	13.37	10.93	7.78	6.20	5.15	3.63	2.63
6	26.59	17.70	18.19	13.81	11.23	7.84	6.34	5.07	3.64	2.68
7	24.11	15.97	16.73	12.70	10.33	7.58	5.83	4.68	3.25	2.41
8	24.29	15.89	16.52	12.80	10.33	7.67	5.79	4.61	3.32	2.41
9	24.35	15.89	16.69	12.83	10.20	7.58	5.74	4.58	3.27	2.38
10	20.29	15.93	16.49	12.80	10.20	7.53	5.76	4.61	3.26	2.41

**Fig. 1:**
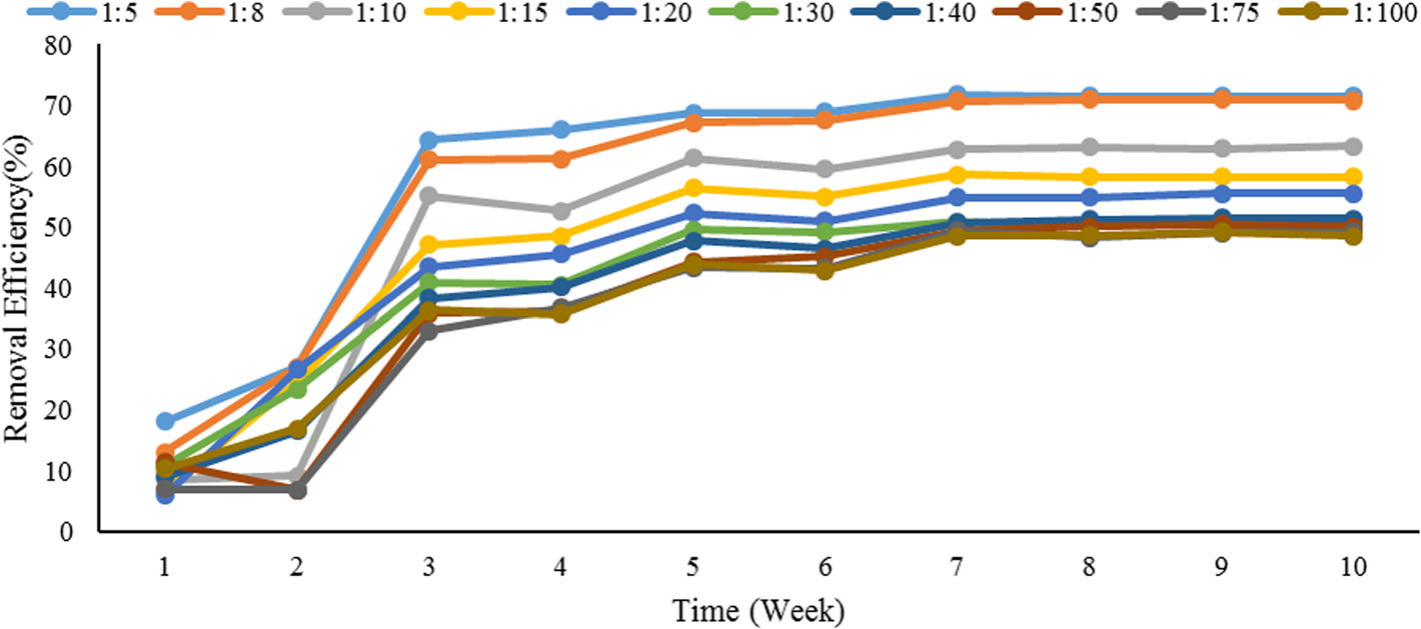
Trend of TPH removal during composting period

Trend of TPH removal during the composting process is shown in Fig. 1. The lowest TPH removal was observed at 1:100 (48.53%) mixing ratio.

Removal efficiency of TPH at mixing ratios of less than 1:10 was more than 60% and for mixing ratios of more than 1:10 was 48.53 to 58.34%.

### Effect of organic carbon (OC)

**Fig. 2:**
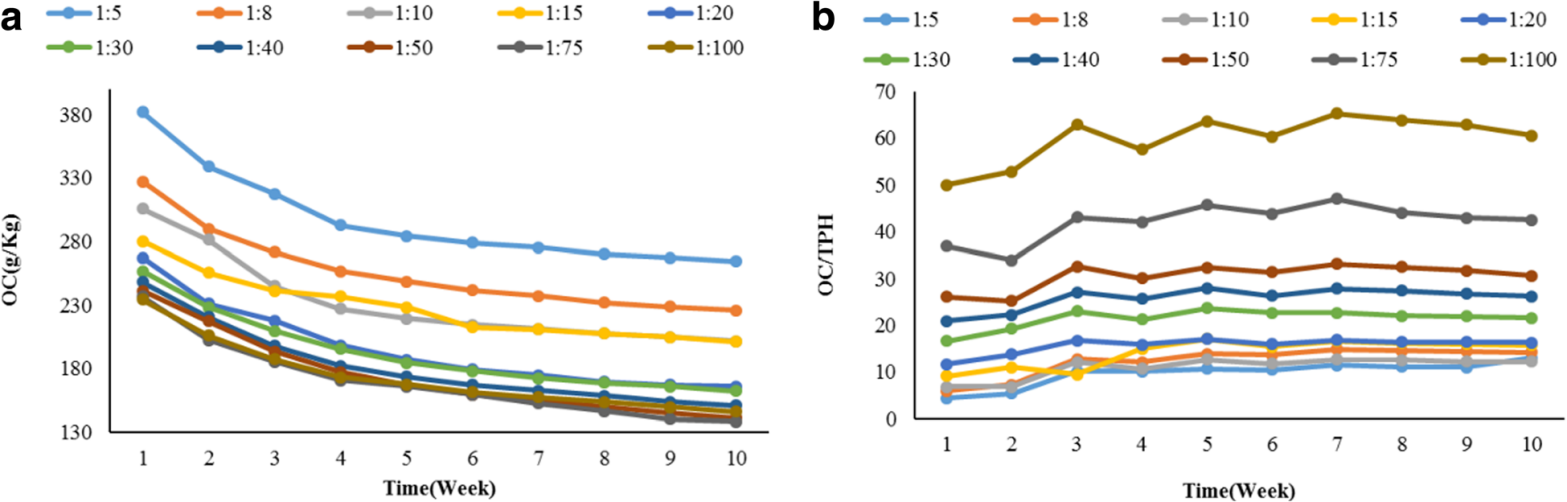
Trend of (**a**) OC consumption and **b** OC: TPH changes during composting period

The trend of OC changes in all reactors during the reaction is shown in Fig. 2 (a). the results showed that the OC content in all reactors has gradually declined. According to the results of this study, TPH and organic carbon variations are almost the same (Figs. 1 and 2 (a)).

### Monitoring parameters

**Fig. 3:**
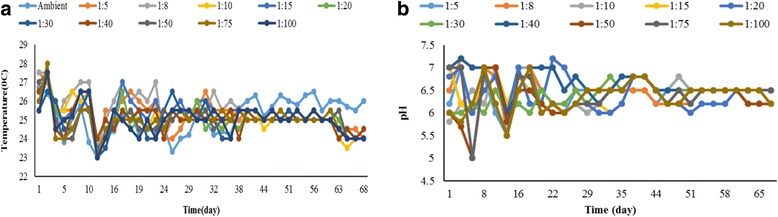
**a** Temperature and **b** pH profiles during composting for removing TPH

The temperature and pH monitoring during the 10-week of experiments are shown in Fig. 3. According to the results, at the beginning of the process, the reactors temperature was above the ambient temperature up to the 42 days (6^th^ Week).

The maximum and minimum temperatures of ambient air during the experiments were 26.5 and 23 °C and in the composting reactors were 28 and 23 °C. In addition, based on the results, the experimental range pH was 6.7 to 7.2 at the initial stages and then drop to nearly 5.5 after 2 weeks and then, after 2 weeks, pH was increased.

### Changes of other important factors in composting

**Fig. 4:**
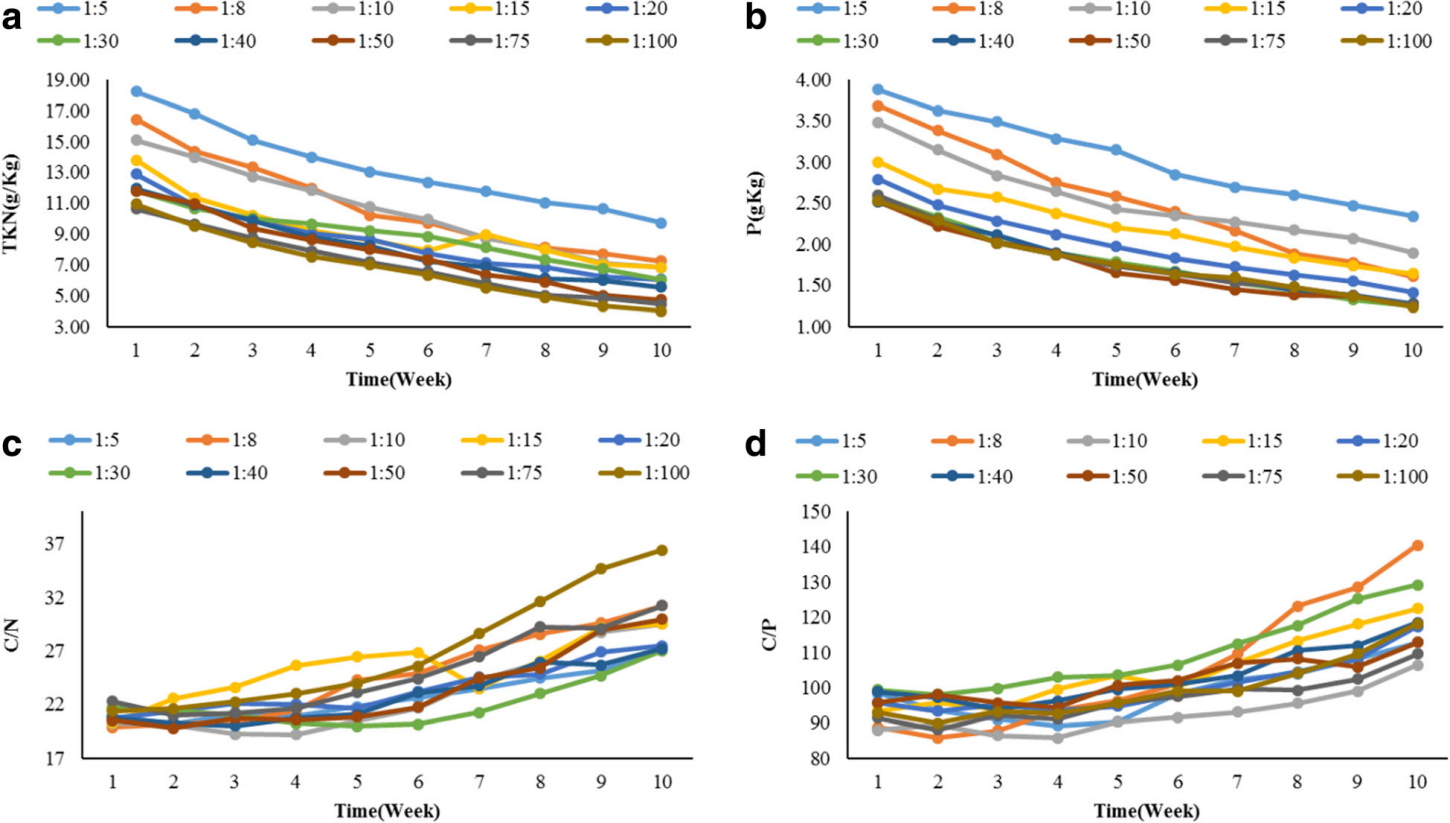
Trend of changes in parameters during the composting process **a** TKN **b** TP **c** C: N and **d** C: P

The variations of the TKN, TP, C: N and C: P in all the reactors for the in-vessel composting process are presented in Fig. 4. The maximum and minimum consumption of carbon, nitrogen, phosphorus during composting in 10 weeks occurred in mixing ratio of 1:5 (117.16 g) and 1:15 (78.92 g), 1:8 (9.22 g) and 1:30 (5.84 g) and 1:8 (2.08 g) and 1:100 (1.24 g), respectively. According to the result of oily sludge, biodegradation in one of the former study under different nutrient conditions (C: N: P ratio of 100:1.74:0.5) showed that after 30 days of treatment maximum removal efficiency of TPH was about 51% and in this research C: N: P ratio in the initial were about 100:5:1 and maximum and minimum removal efficiency after the same time were about 43.95% (1:100 mixing ratio) to 68.80% (1:5 mixing ratio).

### Abiotic degradation and volatilization

Based on the results, total volatilization loss after 10^th^ week with and without HgCl_2_ were 1.22 and 2.00%, respectively. Therefore, the role of abiotic and volatilization loss is very low and negligible.

### Kinetic of TPH removal

The results of kinetic models and half- lives are shown in Table 4. First order kinetic models showed the rate of biodegradation in 1:5 mixing ratio (k1 = 0.0657d^−1^; half-life = 10.55d) was 50% faster than 1:100 mixing ratio (k = 0.0338d^−1^ half-life = 20.51d). Based on second order, the rate of TPH degradation in 1:5 mixing ratio (k2 = 0.0038 d^−1^; half-life = 3.08d) was nearly three times faster than 1:100 mixing ratio (k2 = 0.0238 d^−1^; half-life = 8.96d). The R2 values for the second order models were higher than first order model and indicate the stronger the correlation (Table 4).

**Table 4:** Kientic models for biodegradation of TPH using in-vessel composting

Mixing ratios	First order rate constant, k_1_ (d^−1^)	Half-life, t_1/2_ (d)	R^2^	Second order rate constant, k_2_ (gmg ^−1^d^−1^)	Half-life, t_1/2_ (d)	R^2^
1:5	0.0657	10.55	0.7827	0.0038	3.08	0.8682
1:8	0.0612	11.33	0.7758	0.0052	3.52	0.8488
1:10	0.0512	13.54	0.7525	0.0045	4.94	0.8006
1:15	0.0466	14.87	0.8204	0.0056	5.81	0.8450
1:20	0.0393	17.64	0.7871	0.0062	7.03	0.8480
1:30	0.0350	19.80	0.7670	0.0077	8.41	0.8133
1:40	0.0362	19.15	0.8219	0.0104	8.11	0.8675
1:50	0.0370	18.73	0.8451	0.0133	8.14	0.8879
1:75	0.0352	19.69	0.8507	0.0181	8.61	0.8878
1:100	0.0338	20.51	0.8320	0.0238	8.96	0.8770

## Discussion

### Characteristics of the acidic sludge and immature compost

As it clears more than 50% of acidic sludge mass containing by TPH, and pH in this type of sludge is extremely acidic (pH = 1.35). Which means the waste petroleum compounds and low pH are the main concerns related to environmental pollution. TPH contents in oily sludge in the previous studies are the range of 5 to 86.2% by mass, but more frequently in the range of 15 to 50% [10, 14, 31].

The C: N and C: P for the acidic sludge and immature compost illustrate that the ratio of C: N in the immature compost represents that the high amount of nitrogen in the composition of municipal waste of Tehran is mainly because of the presence of vegetables and plants.

Difference between concentrations of the elements in the acidic sludge showed that the high concentrations of the elements in this type of waste may have originated from the contact between oil of the engine and main source of oil. Previous studies on the elements contained in the oily sludge indicate high concentrations of the elements [14, 21, 31].

### TPH removal in composting reactors

According to obtained results, the maximum removal efficiency of TPH was actually occurred in the first 3 weeks which represents a further decomposition of the hydrocarbons (mainly saturated fractions) at the early stage of composting [32, 33]. Reducing the rate of biodegradation in the next weeks can be associated with the presence of non-biodegradable TPH [15, 21, 34].

Removal efficiency of TPH at mixing ratios of less than 1:10 was more than 60% and for mixing ratios of more than 1:10 was 48.53 to 58.34%, therefore mixing ratio have significant effects for removing organic compounds and TPH. Previous studies also reach the similar results [19, 21, 35]. However, the results showed that the mixing ratios more than 1:10 not only requires a lot of compost to reduce the degree of contamination but also the removal efficiency are not significantly increased in comparison with ratios of less than 1:10. Whereas the results of previous studies indicated that with further increase in organic amendment, removal efficiency of TPH is reduced [36, 37].

### Effect of organic carbon (OC)

The results showed that the OC content in all reactors has gradually declined and this decrease was caused by the loss of organic carbon through biological degradation. According to the results of this study, TPH and organic carbon variations are almost the same (Figs. 1 and 2 (a)). The amendment material used for the supply of OC sources should not be utilized as carbon competitor for energy sources. In this research, the increase of OC: TPH ratio showed that TPH removal was faster than carbon consumption (Fig. 2 (b)).

### Monitoring parameters

The results related to the monitoring illustrated that there is a strongly correlated between temperature and the rate of biological reactions in the composting process [38] and then increasing the temperature reflects the activity of microorganisms in the composting process [39]. Decreasing the temperature after 6 weeks indicates a depletion of the biodegradable material [28]. Possibly reactor temperatures increase extremely due to the small scale effect of reactors used in this study.

Based on results attributed to the temperature (Figs. 1 and 3 (a)), TPH removal efficiency was very slow and limited after reducing the reactor temperature below the ambient temperature. Also, it was found that reactor temperature is an effective factor for removing TPH by composting [21, 22, 40]. The pH is another important parameter in the process of composting and microbial activity. In this study pH experimental range was 6.7 to 7.2 at the initial stages and then drop to nearly 5.5 after 2 weeks. Degradation of organic matter and production of carbon dioxide and organic acids during the composting in the reactors are the main reason of pH drop [28, 38, 41]. Finally, after 2 weeks, pH was increased and this increasing maybe caused by degradation on organic acids along mineralization of nitrogen and after 4 weeks (Fig. 3 (b)) pH remained stable in the range of 6.5 to 7.

### Changes of other important factors in composting

In the composting process there are some important factors for biodegradation such as: C:N, and C:P ratios, these ratios are the key factors for microbial activities along composting process [42, 43]. It is obvious that amount of carbon required for biodegradation of TPH depends on the amount of nitrogen and phosphorus [44]. Increasing the amount of carbon and consequently reducing the nitrogen and phosphorus content during the decomposition of organic matters can lead to reducing a large number of bacteria population.

Based on the results (Fig. 4 (a and b)), nitrogen and phosphorus content in the compost reactors during the time decreased and fluctuations of this reduction at all the reactors were in the similar pattern. The nitrogen and phosphorus consumptions in the final weeks of the process are less than the initial weeks, this implies that the biological activity is involved in the degradation and consumption of petroleum hydrocarbons. Increased levels of C: N, and C: P during the composting process (Fig. 4 (c and d)) represents the amount of consumption of nitrogen and phosphorus from organic carbon [21].

### Abiotic degradation and volatilization

According to the results from abiotic degradation and volatilization, the role of abiotic and volatilization loss is very small and negligible. The operational temperature in this study (mesophilic range) was lower than the extent that could have a significant impact on the loss of TPH by volatilization mechanism [35, 45]. Therefore, microbial activity in composting process was the main reason for TPH removal.

### Kinetic of TPH removal

Data obtained from the kinetics could be examined to realize the dynamics of the reactions [46–48]. In this study kinetic analysis as an important factor for degradation of TPH removal is based on first and second orders were studied. In the previous studies kinetic modeling for hydrocarbon biodegradation has been described [49–53]. The information on kinetic of TPH biodegradation by in-vessel composting is so important because the remaining concentration of contaminant can be identified at any time and on the other hand prediction of the process would be done with kinetic analysis.

First order kinetic models showed the rate of biodegradation in 1:5 mixing ratio was 50% faster than 1:100 mixing ratio. Based on second order, the rate of TPH degradation in 1:5 mixing ratio was nearly three times faster than 1:100 mixing ratio. The R2 values for the second order models were higher than first order model and indicate the stronger the correlation (Table 4). Therefore, shorter half-life values and stronger R2 values (more than 0.8006) indicates faster degradation rate of TPH. The results of this study is similar to previous studies in order to removal of the biodegradable TPH [52, 54].

## Conclusion

The results of this study showed that the combination of acidic sludge that was produced by re-refinery industries and composting materials could lead to a significant reduction of TPH. Microorganisms played an important role in this process. According to the results, mixing ratio (sludge to compost) less than 10: 1 could be removed TPH significantly (more than 63%). In spite of TPH residual was higher than existing environmental standards, but the aerated in-vessel composting by the addition of immature compost as an amendment was an option that could be considered as a feasible method for reduction of TPH. Therefore, final product of this process was well decontaminated and pH of that was natural. However, it is recommended that the final product has been disposed in the sanitary landfill.

## Abbreviations

C: Carbon
N: Nitrogen
OC: Organic carbon
P: Phosphorus
TKN: Total Kjeldahl nitrogen
TPH: Total petroleum hydrocarbon
