# Erratum to: Biodegradation of total petroleum hydrocarbons from acidic sludge produced by re-refinery industries of waste oil using in-vessel composting

**DOI:** 10.1186/s40201-017-0275-1

**Published:** 2017-06-20

**Authors:** Alireza Asgari, Ramin Nabizadeh, Amir Hossein Mahvi, Simin Nasseri, Mohammad Hadi Dehghani, Shahrokh Nazmara, Kamyar Yaghmaeian

**Affiliations:** 1 Department of Environmental Health Engineering, School of Public Health, Tehran University of Medical Sciences, Tehran, Iran; 2 Center for Solid Waste Research (CSWR), Institute for Environmental Research(IER), Tehran University of Medical Sciences (TUMS), Tehran, Iran; 3 Center for Water Quality Research (CWQR), Institute for Environmental Research (IER), Tehran University of Medical Sciences (TUMS), Tehran, Iran

## Erratum

Following publication of this article [1], it has come to our attention that the affiliations were captured as:

^1^ Center for Solid Waste Research (CSWR), Institute for Environmental Research (IER), Tehran University of Medical Sciences (TUMS), Tehran, Iran.

^2^ Department of Environmental Health Engineering, School of Public Health, Tehran University of Medical Sciences, Tehran, Iran.

^3^ Center for Water Quality Research (CWQR), Institute for Environmental Research (IER), Tehran University of Medical Sciences (TUMS), Tehran, Iran

However, the correct affiliations should be as following:

Alireza Asgari

Department of Environmental Health Engineering, School of Public Health, Tehran University of Medical Sciences, Tehran, Iran, Email: asgari.alirezaa@gmail.com

Ramin Nabizadeh

Department of Environmental Health Engineering, School of Public Health, Tehran University of Medical Sciences, Tehran, Iran, Email: rnabizadeh@gmail.com

Amir Hossein Mahvi

Department of Environmental Health Engineering, School of Public Health, Tehran University of Medical Sciences, Tehran, Iran

Center for Solid Waste Research (CSWR), Institute for Environmental Research(IER), Tehran University of Medical Sciences (TUMS), Tehran, Iran, Email: ahmahvi@yahoo.com

Simin Nasseri

Department of Environmental Health Engineering, School of Public Health, Tehran University of Medical Sciences, Tehran, Iran

Center for Water Quality Research (CWQR), Institute for Environmental Research (IER), Tehran University of Medical Sciences (TUMS), Tehran, Iran, Email: naserise@tums.ac.ir

Mohammad Hadi Dehghani

Department of Environmental Health Engineering, School of Public Health, Tehran University of Medical Sciences, Tehran, Iran, Email: dehghanihadi6@gmail.com

Shahrokh Nazmara

Department of Environmental Health Engineering, School of Public Health, Tehran University of Medical Sciences, Tehran, Iran, Email: snazmara@gmail.com

Kamyar Yaghmaeian

Center for Solid Waste Research (CSWR), Institute for Environmental Research(IER), Tehran University of Medical Sciences (TUMS), Tehran, Iran

Department of Environmental Health Engineering, School of Public Health, Tehran University of Medical Sciences, Tehran, Iran

*Corresponding Author: Email: kyaghmaeian@gmail.com, Tel.: +98 912 3311992
